# Long-Term Use of Riluzole Could Improve the Prognosis of Sporadic Amyotrophic Lateral Sclerosis Patients: A Real-World Cohort Study in China

**DOI:** 10.3389/fnagi.2016.00246

**Published:** 2016-10-24

**Authors:** Lu Chen, Xiaolu Liu, Lu Tang, Nan Zhang, Dongsheng Fan

**Affiliations:** Department of Neurology, Peking University Third HospitalBeijing, China

**Keywords:** amyotrophic lateral sclerosis, Chinese cohort, prognosis, real-world study, riluzole

## Abstract

**Objectives:** To investigate the effectiveness of riluzole in a long-term follow-up of cohort with sporadic amyotrophic lateral sclerosis (ALS) in a real-world study.

**Methods:** Patients with ALS between 2007 and 2013 were followed up every 3 months. Survival and tracheotomy were predefined as primary outcome measures. The cumulative defined daily dose (cDDD) of riluzole was estimated. The patients in the riluzole group were classified into 1 of 3 subgroups according to the cDDD quartiles. Survival was analyzed using Kaplan–Meier and Cox regression analysis.

**Results:** Of the 1,540 ALS patients, 415 (26.9%) used riluzole, and the remainder did not. In the riluzole group, the age at onset was greater (*p* = 0.016), the diagnostic delay was shorter (*p* < 0.0005), the body mass index (BMI) was higher (*p* < 0.0005), and the scores for both the functional rating scale (FRS) and the revised FRS (FRS-R) were higher (both *p* < 0.0005) than those of the control group. The median cDDD of riluzole was 28 (2,800 mg). Although Kaplan–Meier analysis did not reveal a significant difference between the two groups (*p* = 0.780), it showed that the prognosis of the beyond quartile 3 subgroup [cDDD ≥ 168 (16,800 mg)] was significantly better than that of the other groups [adjusted HR 0.488 (0.320–0.746), *p* = 0.001].

**Conclusion:** In China, older ALS patients and patients who had a higher BMI, shorter diagnostic delay, and higher FRS or FRS-R scores were more likely to use riluzole. Long-term use of riluzole was associated with a better prognosis for ALS patients, whereas short-term use had little effect on survival.

## Introduction

Amyotrophic lateral sclerosis (ALS) is a fatal neurodegenerative disease that is characterized by the progressive degeneration of upper and lower motor neurons. The typical clinical features of this disease include limb paralysis, muscle atrophy, dysphagia, dysarthria, shortness of breath, and respiratory failure (Mandrioli et al., 2006; Chiò et al., 2009; Robberecht and Philips, 2013). In most studies, the reported median survival time for ALS patients is 3–5 years, and riluzole has been found to be effective in improving the prognosis of ALS patients (Mandrioli et al., 2006; Sabatelli et al., 2008; Chiò et al., 2009; Wijesekera et al., 2009; Kiernan et al., 2011; Miller et al., 2012; Lee et al., 2013). Because ALS treatment is not covered by medical insurance in China and because of the high cost of riluzole, the percentage of Chinese sporadic ALS patients using riluzole was only approximately one third, a fraction that is much lower than that reported in other studies (Chen et al., 2015). It is important to determine whether riluzole is effective as a real-world treatment for Chinese ALS patients and to improve patient counseling and the design of clinical trials.

Compared to clinical trials in which the phenotype and severity of a disease are narrowly defined, cohort studies, which include patients across the full clinical spectrum of a disease, maybe a superior “real-world” representation of the disease (Cetin et al., 2015). Although riluzole has been used in ALS patients for many years, its effectiveness has seldom been confirmed in real-world studies. In this eight-year prospective, clinic-based cohort study, we followed ALS patients of different phenotypes and demonstrated an effect of long-term riluzole use in our patients.

## Materials and Methods

### Protocol Approvals, Registrations, and Patient Consent

This study was approved by the institutional ethics committee of Peking University Third Hospital (PUTH; IRB00006761). The study group obtained written informed consent from each patient before they participated in the study.

### Subjects and Definitions

All patients with a diagnosis of ALS from January 2007 to December 2013 were recruited, screened, and followed up every 3 months. Each patient was independently interviewed and examined by two board-certified neurologists from the study group who had experience with motor neuron diseases. Patients were categorized with limb-onset ALS, bulbar-onset ALS, flail-arm syndrome (FAS), progressive muscular atrophy (PMA), or primary lateral sclerosis (PLS) according to the site of onset and clinical features (Wijesekera et al., 2009; Kiernan et al., 2011). For all cases, baseline demographic details, clinical data, the functional rating scale (FRS) score and the revised FRS (FRS-R) score were collected on the patient’s first visit and at follow-up visits. Survival and tracheotomy were predefined as primary outcome measures. Information about the use of riluzole was acquired from hospital and pharmacy medical records and during follow-up visits. The censoring date for the survival data was January 31, 2015. Patients lost to follow-up were censored at the last known living date.

Patients were diagnosed and classified according to the Airlie House diagnostic criteria (Brooks et al., 2000). Because patients with pure lower motor syndromes could not be classified using these established criteria, they were classified into an additional category of suspected ALS (Wijesekera et al., 2009). Patients in the suspected ALS category were classified into the PMA, limb-onset ALS or FAS categories based on their subsequent clinical evaluations.

In the database, “Use of riluzole” was defined as treatment with riluzole (50 mg) twice per day for longer than 2 weeks. According to previous studies, the dose of riluzole administered to a 70-kg adult in 1 day (100 mg) was defined as 1 defined daily dose (DDD), which has been recommended by the World Health Organization as a unit to use to assess the standard dose of drug. The cumulative DDD (cDDD), which is a measure of the duration of drug use, was estimated as the sum of the dispensed DDD of riluzole and was analyzed with respect to ALS prognosis (Lin et al., 2015). The patients were divided into the control group or the riluzole group according to their use of riluzole. In the riluzole group, the cDDD of riluzole was estimated from the medical record of each patient. The patients in the riluzole group were then classified into 1 of 3 subgroups according to the cDDD: The below quartile 1 group, the quartile 1 through 3 group, or the beyond quartile 3 group. Differences between the subgroups were also analyzed. “Lost to follow-up” was defined as a change in the telephone number of a patient or a patient’s refusal to participate in follow-up evaluations more than three times. “Diagnostic delay” was defined as the time from symptom onset to a confirmed diagnosis of ALS made by a board-certified neurologist. “Contact with pesticides” was defined as selling, delivering, or spraying pesticides at least one time in daily life. “Alcohol abuse” was defined as consuming an alcoholic drink more than twice a week for more than 1 year. Residence was categorized as rural or urban.

### Statistical Analysis

The study group collected paper copies of case report forms from the clinic weekly. Data were double-entered and double-checked by independent investigators. The statistical analysis was first conducted using the total group of patients and then using subgroups defined according to sex, phenotype, age at onset, and dose of riluzole. All variables were analyzed using descriptive statistics, including the mean, median, 95% confidence interval (CI), and inter-quartile range (IQR). Significance was tested at the 5% level, and all analyses were performed using the SPSS V.16.0 software package (SPSS, Chicago, IL, USA). Q–Q plots were used to determine whether the data were normally distributed. Some data that were not normally distributed were normalized by a log transformation. For continuous data that were normally distributed, parametric tests [one-way analysis of variance (ANOVA) or Student’s *t*-test] were used to compare subgroups, and for categorical variables, non-parametric tests (χ^2^ test, Fisher’s exact test, Kruskal–Wallis one-way ANOVA by ranks, or Mann–Whitney *U* test) were used. Kaplan–Meier analysis was used to describe the survival curve. Covariates were analyzed using the log-rank test and Cox regression analysis. When the survival times of patients with the different phenotypes were examined, the PLS group was excluded from the survival analysis because no patients in that group had died. FRS and FRS-R scores were collected on the first visit and at every follow-up visits, but only the first FRS and FRS-R scores were used in the analysis as the baseline score and as a factor of prognosis in the Cox regression model.

## Results

During the study period, 1,540 individual sporadic ALS patients were identified. Of these, 1,125 (73.1%) patients were in the control group, and 415 (26.9%) patients were in the riluzole group. The characteristics of the different groups are shown in **Table 1**. Till January 31, 2015, 662 patients reached the end point and 157 were in the riluzole group. The percentage of patients who reached the end point was higher in the control group (44.9%) than in the riluzole group (37.8%; *p* = 0.015). The percentage of patients who lived in an urban area was higher in the riluzole group (70.9%) than in the control group (60.3%; *p* < 0.0005). Spearman correlation analysis revealed that living in a rural area was correlated with lower FRS (*r*_p_ = -0.072, *p* = 0.016) and FRS-R scores (*r*_p_ = -0.071, *p* = 0.018).

**Table 1:** Demographics of Chinese sporadic ALS patients who did or did not use riluzole.

	Patients who used riluzole	Patients who did not use riluzole	*p*-value
Number of patients (%)	415 (26.9)	1125 (73.1)	
Age at onset [mean (95% CI), years]	51.1 (50.0–52.2)	49.5 (48.8–50.2)	0.016
Gender ratio (M:F)	1.55:1	1.76:1	0.284
% of cases of different phenotypes			0.036
Limb-onset ALS	77.1	71.0	
Bulbar-onset ALS	15.9	16.5	
FAS	4.8	8.6	
PMA	1.9	3.0	
PLS	0.2	0.8	
Diagnostic delay [median inter-quartile range (IQR), months]	11.0 (11.0)	15.0 (17.0)	<0.0005
BMI [mean (95% CI), kg/m^2^]	23.3 (23.0–23.6)	22.6 (22.4–22.9)	<0.0005
% of cases that reached a categorization of laboratory-supported probable at presentation	81.0	74.9	0.015
Functional rating scale (FRS) score [median (IQR)]	34.0 (7.0)	32.0 (9.0)	<0.0005
Revised FRS (FRS-R) score [median (IQR)]	42.0 (7.0)	40.0 (9.0)	<0.0005


*ALS, amyotrophic lateral sclerosis; FAS, flail arm syndrome; PMA, progressive muscular atrophy; PLS, primary lateral sclerosis; BMI, body mass index; CI, confidence interval.*

### Subgroup Analyses

#### Subgroups Based on Riluzole cDDD

In the riluzole group, the cDDD of one quartile was 28 (2,800 mg), and the cDDD of three quartiles was 168 (16,800 mg). There were 182 patients in the below quartile 1 group, 118 patients in the quartile 1 through 3 group, and 115 patients in the beyond quartile 3 group. The mean age at onset was 50.6 years old in the below quartile 1 group (95% CI 48.9–52.3), 51.8 years old in the quartile 1 through 3 group (95% CI 49.7–53.9), and 51.3 years old in the beyond quartile 3 group (95% CI 49.1–53.5). The mean age at onset did not significantly differ between the subgroups (*p* = 0.659). The M:F ratio was 1.49:1 in the below quartile 1 group, 1.51:1 in the 1 through 3 group, and 1.67:1 in the beyond quartile 3 group. Differences in the M:F ratio between the three subgroups were not significant (*p* = 0.902). The body mass index (BMI) of the beyond quartile 3 group [23.8 kg/m^2^ (95% CI 23.2–24.3)] was higher than that of the quartile 1 through 3 group [22.9 kg/m^2^ (95% CI 22.3–23.5)], but pair-wise comparisons between the other subgroups did not reveal significant differences. The median diagnostic delay did not significantly differ between the three subgroups [the below quartile 1 group, 11.0 months (IQR 10); the quartile 1 through 3 group, 11.0 months (IQR 7.75); the beyond quartile 3 group, 12.0 months (IQR 11.75)] (*p* = 0.160). The median FRS score was 34.0 (IQR 6) in the below quartile 1 group, 34.5 (IQR 7) in the quartile 1 through 3 group, and 34.0 (IQR 7) in the beyond quartile 3 group. Neither the FRS score (*p* = 0.733) nor the FRS-R score differed between the groups [the below quartile 1 group, 42.0 (IQR 6); the quartile 1 through 3 group, 42.0 (IQR 8), the beyond quartile 3 group, 42.0 (IQR 7)] (*p* = 0.730).

#### Gender-Based Subgroups

Among the male patients, 252 (26.0%) used riluzole, and 718 (74.0%) did not. The mean age at onset of the riluzole group was 52.0 years old (95% CI, 50.6–53.4), which was significantly older than that of the control group [49.8 years old (95% CI, 49.0–50.7)] (*p* = 0.011). The BMI of the riluzole group [23.4 kg/m^2^ (95% CI, 23.1–23.8)] was significantly higher than that of the control group [22.6 kg/m^2^ (95% CI, 22.3–22.9)] in the male patients (*p* = 0.001). The median diagnostic delay was 14.0 months (IQR 15.0), and the difference between the riluzole group and the control group was also significant [riluzole group, 10.5 months (IQR 9.0); control group, 16.0 months (IQR 17.0)] (*p* < 0.0005). The FRS [34.0 (IQR 7.0)] and FRS-R scores [42.0 (IQR 7.0)] of the riluzole group were higher than those of the control group [FRS, 33.0 (IQR 8.0), *p* = 0.004; FRS-R, 41.0 (IQR 8.0), *p* = 0.002].

Among the female patients, 163 (28.6%) used riluzole, and 407 (71.4%) did not. The mean age at onset in the control [48.9 years old (95% CI, 47.7–50.1)] and riluzole groups [49.8 years old (95% CI, 48.0–51.6)] did not significantly differ (*p* = 0.425). There were no significant differences in BMI between the riluzole group [23.0 kg/m^2^ (95% CI, 22.5–23.5)] and the control group [22.6 kg/m^2^ (95% CI, 22.2–23.0)] (*p* = 0.274). The median diagnostic delay of the female patients was 14.0 months (IQR 15.0), and the difference between the riluzole group and the control group was significant [riluzole group, 12.0 months (IQR 12.0); control group, 15.0 months (IQR 16.0)] (*p* < 0.0005). The FRS [34.0 (IQR 7.0)] and FRS-R scores [42.0 (IQR 8.0)] of the riluzole group were higher than those of the control group [FRS, 31.0 (IQR 10.0), *p* < 0.0005; FRS-R, 39.0 (IQR 10.0), *p* < 0.0005].

#### Phenotype-Based Subgroups

The percentage of patients who used riluzole was 28.6% (320/1,118) in the limb-onset ALS, 26.2% (66/252) in the bulbar-onset ALS, 17.1% (20/117) in the FAS, and 19.0% (8/42) in the PMA. The proportion of riluzole users was not significantly different between the limb-onset ALS and the bulbar-onset ALS (*p* = 0.244), but it was higher in limb-onset ALS than in the FAS and PMA. There were ten patients in the PLS, and only one used riluzole. The proportion of riluzole users significantly differed between the phenotypes (*p* = 0.036). The age at onset was significantly greater in the riluzole group [49.9 years old (95% CI, 48.6–51.1)] than in the control group [48.3 years old (95% CI, 47.5–49.1)] only in the limb-onset ALS (*p* = 0.036). Differences in BMI were observed only in the bulbar-onset ALS (riluzole group, 23.03 kg/m^2^; control group, 21.79 kg/m^2^; *p* = 0.011) and the PMA (riluzole group, 25.11 kg/m^2^; control group, 21.82 kg/m^2^; *p* = 0.030). Compared with the diagnostic delay of the control group, that in the riluzole group was significantly shorter when this parameter was analyzed in the limb-onset ALS [riluzole group, 11.0 months (IQR 10.0); control group, 15.0 months (IQR 16.0); *p* < 0.0005], the bulbar-onset ALS [riluzole group, 10.0 months (IQR 8.0); control group, 13.0 months (IQR 12.0); *p* < 0.0005], and the FAS [riluzole group, 12.0 months (IQR 14.75); control group, 18.0 months (IQR 27.0); *p* = 0.028]. The FRS and FRS-R scores were higher in the riluzole group than in the control group in the limb-onset ALS (FRS, *p* = 0.002; FRS-R, *p* = 0.001), bulbar-onset ALS (FRS, *p* < 0.0005; FRS-R, *p* < 0.0005), and FAS (FRS, *p* = 0.010; FRS-R, *p* = 0.010).

#### Age-Based Subgroups

In order to analyze the difference between riluzole and control groups in different age groups, we divided the patients into three age groups according to the quartiles of age at onset: Age <42.0 years old group, age between 42.0 and 58.0 years old group, and age ≥58.0 years old group. The percentage of patients who used riluzole was 26.1% (96/368) in the age <42.0 years old group, 23.9% (177/740) in the age between 42.0 and 58.0 years old group, and 32.9% (142/432) in the age ≥58.0 years old group. The proportion of patients who used riluzole was significantly different between the age groups (*p* = 0.004). There was no significant difference in the M:F ratio of the riluzole group and the control group in all the three age groups (in age <42.0 years old group: Riluzole group 1.2 vs. control group 1.5, *p* = 0.194; in age between 42.0 and 58.0 years old group: Riluzole group 1.9 vs. control group 1.6, *p* = 0.244; in age ≥58.0 years old group: Riluzole group 1.8 vs. control group 1.7, *p* = 0.452). The BMI of the riluzole group was significantly higher than that of the control group in the age between 42.0 and 58.0 years old group [riluzole group, 23.5 kg/m^2^ (95%CI 23.1–24.0); control group, 22.8 kg/m^2^ (95%CI 22.5–23.2); *p* = 0.016] and age ≥58.0 years old group [riluzole group, 23.0 kg/m^2^ (95%CI 22.5–23.6); control group 22.3 kg/m^2^ (95%CI 21.9–22.8); *p* = 0.016]. The median diagnostic delay time in the riluzole group was shorter than in the control group in all three age groups [in age <42.0 years old group: Riluzole group, 12.0 months (IQR 11.0) vs. control group, 16.0 months (IQR 21.75); in age between 42.0 and 58.0 years old group: Riluzole group, 11.0 months (IQR 10.75) vs. control group, 15.0 months (IQR 17.0); in age ≥58.0 years old group: Riluzole group, 10.0 months (IQR 9.0) vs. control group, 15.0 months (IQR 13.25); all *p* < 0.0005]. The proportions of different phenotypes in the riluzole and the control groups did not show significant difference in all the three age groups (in age <42.0 years old group, *p* = 0.405; in age between 42.0 and 58.0 years old group, *p* = 0.116; in age ≥58.0 years old group, *p* = 0.592). The FRS and FRS-R scores of the riluzole group were significantly higher in the age between 42.0 and 58.0 years old group [FRS: Riluzole group, 34.0 (IQR 6.0) vs. control group, 32.0 (IQR 9.0); FRS-R: Riluzole group, 42.0 (IQR 7.0) vs. control group, FRS-R 40.0 (IQR 10.0); both *p* < 0.0005] and age ≥58.0 years old group [FRS: Riluzole group, 34.0 (IQR 6.0) vs. control group, 32.0 (IQR 8.0), *p* = 0.005; FRS-R: Riluzole group, 42.0 (IQR 7.0) vs. control group, FRS-R 39.0 (IQR 8.0), *p* = 0.003].

### Factors Associated with Survival

**FIGURE 1:**
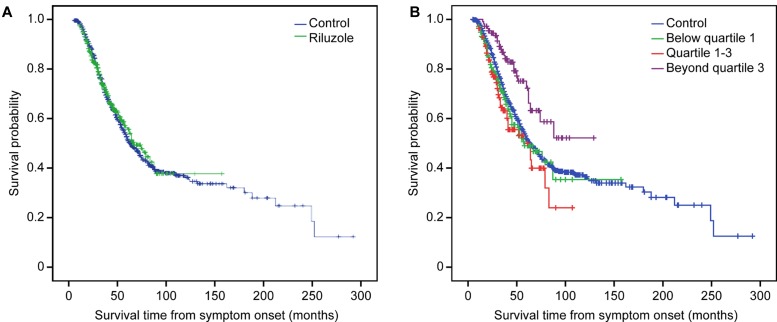
**Kaplan-Meier survival curves.** **(A)** Survival analysis stratified by riluzole; **(B)** Survival analysis stratified by quartiles of dose of riluzole.

The Kaplan–Meier analysis did not reveal any significant differences in the median survival time of the riluzole group [67.0 months (95% CI 54.7–79.3)] and the control group [64.0 months (95% CI 57.8–70.2)] (*p* = 0.780). However, when the prognosis of the subgroups in the riluzole group and the control group were compared, the survival of the beyond quartile 3 subgroup was significantly better than that of the other groups (compared with the control group, *p* = 0.001; compared with the below quartile 1 subgroup, *p* = 0.001; compared with the quartile 1 through 3 subgroup, *p* < 0.0005) (**Figure 1**).

Among the male patients, the median survival time of the riluzole group was 58.0 months (95% CI 46.0–70.0), which did not significantly differ with that of the control group [61.0 months (95% CI 54.3–67.7)] (*p* = 0.395). Subgroup analysis showed that the median survival time of the beyond quartile 3 group was 88.0 months, which was much longer than that of the other subgroups as well as the control group [below quartile 1 group, 55.0 months (95% CI 43.6–66.4), *p* < 0.0005; quartile 1 through 3 group, 41.0 months (95% CI 32.3–49.7), *p* < 0.0005; control group, 61.0 months (95% CI 54.3–67.7), *p* = 0.006]. Among the female patients, the survival times of the riluzole group and the control group did not significantly differ (*p* = 0.192). Subgroup analysis showed that the median survival time also did not differ between the subgroups and the control group (*p* = 0.338).

Because there were not enough cases in the PLS group, the PLS phenotype was excluded from the analysis of different phenotypes. The survival times of the riluzole group and the control group did not significantly differ in any of the phenotypes (limb-onset ALS, *p* = 0.890; bulbar-onset ALS, *p* = 0.788; FAS, *p* = 0.548; PMA, *p* = 0.964). However, analysis of the survival of the different cDDD subgroups within the phenotypes showed that the survival of the beyond quartile 3 group was significantly better than that of the other groups only for patients with limb-onset ALS (compared with the control group, *p* = 0.002; compared with the below quartile 1 group, *p* = 0.001; compared with the quartile 1 through 3 group, *p* < 0.0005).

In the age between 42.0 and 58.0 years old group, median survival was prolonged by 24.0 months (the riluzole group, 83.0 months; the control group, 59.0 months; *p* = 0.024), while in other two age groups there was no significant difference in survival between the riluzole group and the control group. In subgroup analysis of cDDD, the prognosis of the beyond quartile 3 group was significantly better than that of the other groups in the age between 42.0 and 58.0 years old group (compared with the control group, *p* = 0.003; compared with the below quartile 1 group, *p* = 0.017; compared with the quartile 1 through 3 group, *p* = 0.114) and in the age ≥58.0 years old group (compared with the control group, *p* = 0.062; compared with the below quartile 1 group, *p* = 0.001; compared with the quartile 1 through 3 group, *p* = 0.004), and was better than that of the quartile 1 through 3 group in the age <42.0 years old group (*p* = 0.002).

**FIGURE 2:**
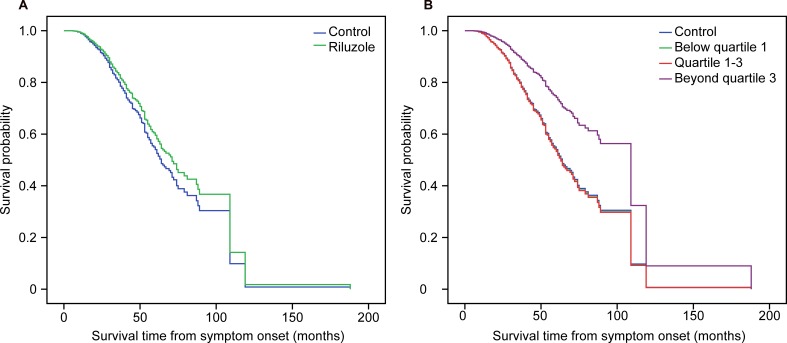
**Survival curves after adjusting for age, gender, BMI, diagnostic delay time, phenotype, Airlie House category at presentation, residence, FRS score, history of smoking or alcohol abuse, contact history of harmful gas or pesticides at the covariate means using Cox regression model.** **(A)** Survival analysis stratified by riluzole; **(B)** Survival analysis stratified by quartiles of dose of riluzole.

In the univariate analysis, the factors related with survival included age at onset [HR 1.044 (1.037–1.052), *p* < 0.0005], gender [female compared with male, HR 0.826 (0.699–0.975), *p* = 0.024], BMI [HR 0.936 (0.908–0.965), *p* < 0.0005], diagnostic delay [HR 0.955 (0.949–0.975), *p* < 0.0005], Airlie House category at presentation [compared with definite ALS: Probable ALS supported by laboratory findings, HR 0.743 (0.562–0.981), *p* = 0.036; others were not significant], phenotype [compared with limb-onset ALS: bulbar-onset ALS, HR 1.448 (1.178–1.779), *p* < 0.0005; others were not significant], contact history with pesticides [HR 1.322 (1.033–1.692), *p* = 0.027], and FRS score [HR 0.975 (0.962–0.988), *p* < 0.0005]. In the multivariate Cox regression model, after adjusting for age, gender, BMI, diagnostic delay, phenotype, Airlie House category at presentation, residence, FRS score, history of smoking or alcohol abuse, and contact history with pesticides, there was no significant difference in survival time between the riluzole group and the control group [adjusted HR 0.855 (0.685–1.068), *p* = 0.167]. However, after adjusting for the factors mentioned above, the subgroup analysis showed that the prognosis of the beyond quartile 3 subgroup was significantly better than that of the control group [adjusted HR 0.488 (0.320–0.746), *p* = 0.001], indicating that the long-term use of riluzole could improve ALS patient survival. Older age at onset, male gender, lower BMI, shorter diagnostic delay from symptom onset, residence in a rural area, and lower FRS score at presentation were also related to poorer ALS patient prognosis in the multivariate Cox regression model (**Figure 2**).

## Discussion

In this study, although the prognosis of the riluzole group and the control group did not significantly differ, the prognosis of the beyond quartile 3 subgroup was much better than that of the other groups even after adjusting for other factors. This result suggests that the long-term use of riluzole may improve the prognosis of ALS patients.

Our data showed that older patients and patients who lived in urban areas and who had a higher BMI, shorter diagnostic delay, higher diagnostic category at presentation, and a higher FRS or FRS-R score were more likely to use riluzole. Based on this result, we can divide the patients who used riluzole into two situations. First, the faster the disease progressed or the more severe the disease, the more willing the patients were to use riluzole. In previous studies, greater age at onset, shorter diagnostic delay from symptom onset and higher diagnostic category at presentation were all related to poorer survival and faster disease progression (Chiò et al., 2009; Chen et al., 2015). However, the shorter diagnostic delay may also be explained by the fact that those who subsequently take riluzole are more motivated to visit the hospital than those who do not (Roche et al., 2012). Second, because a higher BMI and residence in an urban area may be related to better economic conditions, the affordability of the medication for the patients may influence riluzole use. Because residence in a rural area was related to lower FRS and FRS-R scores, the lower FRS and FRS-R scores of the control group may be explained by the greater percentage of patients who lived in a rural area in this group. Because China is still a developing country, there are a large income differences between urban and rural areas, and because ALS treatment is not covered by medical insurance in China, the finding that fewer patients who lived in rural areas could afford the expense of riluzole can be easily explained.

In most previous studies about the prognosis of ALS, riluzole was analyzed as a survival factor, but its daily and cumulative dose has seldom been taken into consideration (Chiò et al., 2002, 2009; Traynor et al., 2003; Kiernan et al., 2011; Lee et al., 2013; Chen et al., 2015). In the prognostic studies, the effect of riluzole on the survival of ALS patients was controversial: Most of the studies found it was related to longer survival, but in our previous study we did not find this tendency (Chen et al., 2015). In a meta-analysis that included four randomized trials of riluzole in a total of 1,477 people with ALS, when the two trials that recruited a homogeneous group of participants were analyzed, riluzole had a significant effect on survival (*p* = 0.039). However, when the third trial that involved more seriously affected and older patients was included in the analysis, the treatment effect was not significant (*p* = 0.056; Miller et al., 2012). Some researchers suggested that riluzole could only slow down and not reverse the degeneration of motor neurons and was therefore less effective in advanced-stage patients (Traynor et al., 2003). Because the age at onset and disease severity are more heterogeneous in a real-world cohort study than in randomized trials, the finding that the prognosis of riluzole group was not better than that of the control group may explained by the fact that older and more severely affected patients were included in the cohort study. However, our subgroup analysis did show that the beyond quartile 3 subgroup (16,800 mg) had a much better prognosis than other groups, even after adjusting for all other factors. Because the DDD of riluzole was 100 mg per day, it appears that the use of riluzole over approximately 6 months may improve ALS patient survival.

It was notable that the effect of long-term riluzole use was found only in the male patients, whereas in the female patients, no significant differences in survival were found between the subgroups. In our previous study, we also found that the male and female patients differed in many aspects, such as the age at onset, survival time, and the prognosis of the different phenotypes. Some investigators have argued that these difference may be explained by metabolic and hormonal differences between males and females (Aksoy et al., 2014; Chen et al., 2015). Because riluzole could only slow down the progression of the disease rather than reverse it, (Traynor et al., 2003) the reduced effectiveness of long-term riluzole use in the female patients may be explained by the significantly longer survival time of female ALS patients, which means the disease itself progresses much more slowly in females than in males.

The lower percentage of riluzole users in the FAS group may be explained by the slower progression of the disease (Chen et al., 2015). The fact that the better prognosis of the beyond quartile 3 group was only observed in the limb-onset ALS group suggests that the mechanism of and response to riluzole differ between the phenotypes. In the first clinical trial of riluzole in 1994, researchers found that riluzole seemed to be more effective in bulbar-onset ALS patients (Bensimon et al., 1994). However, the larger and subsequent clinical trial showed that the difference in the effect of riluzole treatment between bulbar-onset and limb-onset ALS patients might be an artifact of the small sample size of the previous study (Lacomblez et al., 1996). It is interesting that in a population-based study of the Italian patients, riluzole was effective in bulbar-onset ALS patients but had no effect on survival at 12 months in limb-onset ALS patients (Zoccolella et al., 2007). In another population-based study in the Irish people, researchers also found that the bulbar-onset ALS patients benefited more from riluzole treatment, although the reason for this was unknown (Traynor et al., 2003). In our study, however, although the proportion of patients using riluzole showed to be similar in bulbar-onset and limb-onset patients, the prognosis of the riluzole users seemed to be better only in the limb-onset patients. In our previous study, we found that the clinical characteristics of Chinese ALS patients were quite different from that of European and North American patients, and the different effect of riluzole in bulbar-onset and limb-onset patients might also suggest the fact that the features of ALS patients in China are unique (Chen et al., 2015; Huynh and Kiernan, 2015). However, the small sample size for some phenotypes may also have influenced the results of the comparisons.

In the study of Zoccolella et al. (2007) older patients (patients aged > 70 years) benefited more from the treatment of riluzole. In our study, there were only 57 patients older than 70 years old and there was no significant difference in survival between the riluzole and the control group in patients older than 70 years old (32 months for the riluzole group vs. 27 months for the control group; *p* = 0.298). The mean age at onset of ALS patients in China was much younger than that in other countries and the proportion of ALS patients older than 70 years old was much lower than that in Italian (26.1% in Italian patients vs. 3.7% in Chinese patients; Zoccolella et al., 2007; Chen et al., 2015). However, when we divided patients into three groups according to the quartiles of age at onset, median survival was prolonged by 24 months because of the treatment of riluzole in the age between 42.0 and 58.0 years old group, while in age <42.0 and ≥58.0 years old group there was no significant difference in survival between the riluzole group and the control group. In the subgroup analysis, a cDDD of 16,800 mg (about 6-month use) were effective both in the age between 42.0 and 58.0 years old group and in the age ≥58.0 years old group. This result suggests that long-term use of riluzole (cDDD ≥ 16,800 mg) may improve the prognosis of older patients (aged ≥ 42.0 years) and this proved the findings of Zoccolella et al. (2007), although the definition of “older patients” in our study was much younger.

Our study has several limitations. First, although the patients’ use of riluzole was acquired from the hospital and pharmacy medical records as well as from follow-up visits, the number of patients who used riluzole could still have been underestimated. Second, some important data, such as gastrostomy data, the use of other medicine and respiratory status, were absent from our database; the lack of these data could have influenced the data analysis. Third, since the cDDD would be bigger if the patient lived longer, it might be a potential confounder of the dose-dependent effect of riluzole on survival. However, the median survival time of Chinese ALS patients was 71 months (Chen et al., 2015), so a cDDD of 16,800 mg (about 6-month use) might not sufficiently reflect a longer survival of patients. Fourth, due to the limitations of a single-center clinic cohort study, the results may differ from those of a population-based study. The validity of our observations based on this clinical database is strengthened by the findings from a population-based study in Taiwan in which the demographic and clinical features were quite consistent with our data (Lee et al., 2013). Since the importance of population-based studies in defining clinical features and prognostic factors in ALS (Beghi et al., 2006; Logroscino et al., 2008), a nationwide multicenter study or population-based study for Chinese patients should be conducted in the future (Zoccolella et al., 2008; Chen et al., 2015).

In summary, this study showed that in China, older ALS patients and patients who had a higher BMI, shorter diagnostic delay, and higher FRS or FRS-R scores were more likely to use riluzole. The long-term [cDDD ≥ 168 (16,800 mg)] use of riluzole was associated with a better prognosis for ALS patients, whereas short-term use had little effect on survival.

## Author Contributions

DF conceived this study and provided financial support. DF and LC designed the study. XL, LT, and NZ took part in the design of the study and in sample collection. DF, LC, XL, and LT conducted data management. NZ conducted data follow-up. LC and XL undertook data checking. LC, XL, and DF undertook statistical analysis. DF was responsible for project management. LC and DF were responsible for preparing and revising the manuscript. DF and LC had key roles in the study.

## Conflict of Interest Statement

The authors declare that the research was conducted in the absence of any commercial or financial relationships that could be construed as a potential conflict of interest.

## References

[B1] Aksoy D. Cevik B. Solmaz V. Kurt S. G. (2014). Clinical, demographic and prognostic features ofsporadic amyotrophic lateral sclerosis in Northern Turkey. *Int. J. Neurosci.* 124 68–73. 10.3109/00207454.2013.823605 23837674

[B2] Beghi E. Logroscino G. Chiò A. Hardiman O. Mitchell D. Swingler R. (2006). The epidemiology of ALS and the role of population-based registries. *Biochim. Biophys. Acta* 1762 1150–1157. 10.1016/j.bbadis.2006.09.008 17071060

[B3] Bensimon G. Lacomblez L. Meininger V. (1994). A controlled trial of riluzole in amyotrophic lateral sclerosis. ALS/Riluzole Study Group. *N. Engl. J. Med.* 330 585–591. 8302340 10.1056/NEJM199403033300901

[B4] Brooks B. R. Miller R. G. Swash M. Munsat T. L. World Federation of Neurology Research Group on Motor Neuron Diseases. (2000). El Escorial revisited: revised criteria for the diagnosis of amyotrophic lateral sclerosis. *Amyotroph. Lateral Scler. Other Motor Neuron Disord.* 1 293–299. 10.1080/146608200300079536 11464847

[B5] Cetin H. Rath J. Füzi J. Reichardt B. Fülöp G. Koppi S. (2015). Epidemiology of amyotrophic lateral sclerosis and effect of riluzole on disease course. *Neuroepidemiology* 44 6–15. 10.1159/000369813 25571962

[B6] Chen L. Zhang B. Chen R. Tang L. Liu R. Yang Y. (2015). Natural history and clinical features of sporadic amyotrophic lateral sclerosis in China. *J. NeurolNeurosurg. Psychiatry* 86 1075–1081. 10.1136/jnnp-2015-310471 26124198

[B7] Chiò A. Logroscino G. Hardiman O. Swingler R. Mitchell D. Beghi E. (2009). Prognostic factors in ALS: a critical review. *Amyotroph. Lateral Scler.* 10 310–323. 10.3109/17482960802566824 19922118 PMC3515205

[B8] Chiò A. Mora G. Leone M. Mazzini L. Cocito D. Giordana M. T. (2002). Early symptom progression rate is related to ALS outcome: a prospective population-based study. *Neurology* 59 99–103. 10.1212/WNL.59.1.99 12105314

[B9] Huynh W. Kiernan M. C. (2015). A unique account of ALS in China: exploring ethnic heterogeneity. *J Neurol. Neurosurg. Psychiatry.* 86 1051–1052. 10.1136/jnnp-2015-311293 26134851

[B10] Kiernan M. C. Vucic S. Cheah B. C. Turner M. R. Eisen A. Hardiman O. (2011). Amyotrophic lateral sclerosis. *Lancet* 377 942–955. 10.1016/S0140-6736(10)61156-7 21296405

[B11] Lacomblez L. Bensimon G. Leigh P. N. Guillet P. Meininger V. (1996). Dose-ranging study of riluzole in amyotrophic lateral sclerosis. Amyotrophic Lateral Sclerosis/Riluzole Study Group II. *Lancet* 347 1425–1431. 8676624 10.1016/s0140-6736(96)91680-3

[B12] Lee C. T. Chiu Y. W. Wang K. C. Hwang C. S. Lin K. H. Lee I. T. (2013). Riluzole and prognostic factors in amyotrophic lateral sclerosis long-term and short-term survival: a population-based study of 1149 cases in Taiwan. *J. Epidemiol.* 23 35–40. 10.2188/jea.JE20120119 23117224 PMC3700231

[B13] Lin F. C. Tsai C. P. Kuang-Wu Lee J Wu M. T. Tzu-Chi Lee C (2015). Angiotensin-converting enzyme inhibitors and amyotrophic lateral sclerosis risk: a total population-based case-control study. *JAMA Neurol.* 72 40–48. 10.1001/jamaneurol.2014.3367 25383557

[B14] Logroscino G. Traynor B. J. Hardiman O. Chio’ A. Couratier P. Mitchell J. D. (2008). Descriptive epidemiology of amyotrophic lateral sclerosis: new evidence and unsolved issues. *J. Neurol. Neurosurg. Psychiatry* 79 6–11. 10.1136/jnnp.2006.104828 18079297

[B15] Mandrioli J. Faglioni P. Nichelli P. Sola P. (2006). Amyotrophic lateral sclerosis: prognostic indicators of survival. *Amyotroph. Lateral Scler.* 7 211–220. 10.1080/17482960600947648 17127559

[B16] Miller R. G. Mitchell J. D. Moore D. H. (2012). Riluzole for amyotrophic lateral sclerosis/motor neuron disease. *Cochrane Database Syst. Rev.* 14 CD001447. 10.1002/14651858.CD001447 11687111

[B17] Robberecht W. Philips T. (2013). The changing scene of amyotrophic lateral sclerosis. *Nat. Rev. Neurosci.* 14 248–264. 10.1038/nrn3430 23463272

[B18] Roche J. C. Rojas-Garcia R. Scott K. M. Scotton W. Ellis C. E. Burman R. (2012). A proposed staging system for amyotrophic lateral sclerosis. *Brain.* 135(Pt 3), 847–852. 10.1093/brain/awr351 22271664 PMC3286327

[B19] Sabatelli M. Madia F. Conte A. Luigetti M. Zollino M. Mancuso I. (2008). Natural history of young-adult amyotrophic lateral sclerosis. *Neurology* 71 876–881. 10.1212/01.wnl.0000312378.94737.45 18596241

[B20] Traynor B. J. Alexander M. Corr B. Frost E. Hardiman O. (2003). An outcome study of riluzole in amyotrophic lateral sclerosis—a population-based study in Ireland, 1996–2000. *J. Neurol.* 250 473–479. 10.1007/s00415-003-1026-z 12700914

[B21] Wijesekera L. C. Mathers S. Talman P. Galtrey C. Parkinson M. H. Ganesalingam J. (2009). Natural history and clinical features of the flailarm and flail leg ALS variants. *Neurology* 72 1087–1094. 10.1212/01.wnl.0000345041.83406.a2 19307543 PMC2821838

[B22] Zoccolella S. Beghi E. Palagano G. Fraddosio A. Guerra V. Samarelli V. (2007). Riluzole and amyotrophic lateral sclerosis survival: a population-based study in southern Italy. *Eur. J. Neurol.* 14 262–268. 10.1111/j.1468-1331.2006.01575.x 17355545

[B23] Zoccolella S. Beghi E. Palagano G. Fraddosio A. Guerra V. Samarelli V. (2008). Analysis of survival and prognostic factors in amyotrophic lateral sclerosis: a population based study. *J. Neurol. Neurosurg. Psychiatry* 79 33–37. 10.1136/jnnp.2007.118018 17550991

